# Postsurgical Ultrasound Evaluation of Patients with Prosthesis in Acellular Dermal Matrix: Results from Monocentric Experience

**DOI:** 10.1155/2019/7437324

**Published:** 2019-06-16

**Authors:** Laura Ballesio, Alice Casinelli, Silvia Gigli, Cristiana Boldrini, Di Taranto Giuseppe, Antonio Albano, Maria Giuseppina Onesti

**Affiliations:** ^1^Department of Radiological Sciences, “Sapienza” University of Rome, V.le Regina Elena 324, 00161 Rome, Italy; ^2^Department of Plastic and Reconstructive Surgery, “Sapienza” University of Rome, V.le Regina Elena 324, 00161 Rome, Italy

## Abstract

Mastectomy and breast prosthetic reconstruction is the most common surgical treatment for women diagnosed with breast cancer. In the last few years, breast prosthetic augmentation in acellular dermal matrix (ADM) has been introduced. The aim of this study is to present our single-center experience in evaluating the outcome of patients who underwent breast reconstruction in ADM, using ultrasound (US) examination. US follow-up allows evaluating both normal postoperative findings and changes and potential local complications, demonstrating that ADM is a safe option for women candidates for mastectomy.

## 1. Introduction

Approximately 35-40% of women with breast cancer undergo a total mastectomy and most of them are candidates for prosthetic reconstruction. Prosthetic augmentation for reconstructive surgery is a safe surgical option that can be done either at the time of the mastectomy, as immediate breast reconstruction or as a two-stage reconstruction with a tissue expander followed by a permanent implant, as delayed breast reconstruction [1–3].

Although these operations are very common in clinical practice, there may be some side effect related with a limited biocompatibility; the most frequent is capsular contracture. The development of fibrous tissue around the prosthesis represents a physiological mechanism to fix the implant in the breast and to prevent infection and trauma. Moreover, the capsular tissue may stiffen and extend, becoming extremely painful and unaesthetic [4]. Capsular contracture rates in immediate reconstruction has been reported as being between 20% and 40.4%, while rates for delayed reconstruction range from 17% to 26.4% [2]. In addition, radiotherapy, which is part of adjuvant treatment for breast cancer, can also compromise bloody supply to skin leading to a greater risk of tissue damage, infection, capsular reaction, and poor aesthetic result, as reported by Quinn et al. [5].

In the last few years, a novel approach for breast reconstruction has been introduced by using ADM that is an immunologically inert material prepared by xenoplastic or alloplastic cadaveric dermis devoid of cellular elements providing a biological structure useful to embrace tissue ingrowth and improve angiogenesis and cellular regeneration [6, 7]. The use of ADM reduces tissue expansion improving the implant reconstructive process, avoiding mastectomy flap contraction and providing an additional layer of tissue between the skin and the implant and it offers an alternative option for one-stage breast reconstruction [8].

After surgery, a clinical and diagnostic follow-up should be performed in order to recognize postoperative local complications; in this setting, US represents the most used imaging method as it is the most safe, noninvasive, and repeatable technique [7, 9]. A confident diagnosis between normal ADM US features and disease recurrence could be challenging. In this study, we reported our monocentric experience in evaluating the US follow-up findings in a group of patients who underwent breast reconstruction to describe the normal and abnormal imaging findings related with varying degrees of matrix reabsorption.

## 2. Materials and Methods

We performed a prospective study between March 2017 and July 2018, including 27 consecutive patients (age range 29-78 years, mean age 50.3 years) who underwent breast prosthetic augmentation for surgery reconstruction with ADM.

Most patients (19) underwent unilateral mastectomy for breast cancer with delayed breast reconstruction; two women underwent unilateral mastectomy for cancer with immediate prosthetic reconstruction and contralateral breast symmetrisation. Three patients, two of them young sisters with BRCA-1 mutation, underwent a bilateral prophylactic mastectomy and prosthetic reconstruction. Finally, three women received bilateral aesthetic breast augmentation.

All these patients were evaluated after surgery with a follow-up ultrasound (US) examination performed by a Radiologist with 15 years' experience in breast imaging, in an early-, an intermediate-, and a late-phase of the postsurgical convalescence. Therefore, a US evaluation was performed at T0 (1 month), at T1 (3 months), at T2 (6 months), and at T3 (12 months) after surgery, by using a Aplio 500 Ultrasound system (Toshiba, Toshiba Medical System s.r.l.) and a LOGIQ E9 (GE Healthcare) with linear high-frequency transducer (10-15 MHz).

During each US examination, we used a standardized systematic approach, including the evaluation of the visibility and thickness of the biological membrane, the implant morphology, the implant margins, and the presence of mediolateral membrane folds.

In addition, we considered the occurrence of different local complications in course of follow-up, such as the presence of periprosthetic fluid, inhomogeneities of soft tissues, liponecrosis, hematoma, seroma, infection, lymphoceles, and findings of suspected disease recurrence.

Each one of these normal and abnormal findings was observed and recorded; a standard medical report form was proposed and adopted fort each patient at T0 and in the successive evaluations.

Only in one case, during a T2 evaluation on a patient affected by invasive ductal carcinoma, we observed a hypoechoic nodule increased in size from the prior examination; we performed a US-guided biopsy and successive histological examination revealed that it was a residual disease.

## 3. Results

**Figure 1:**
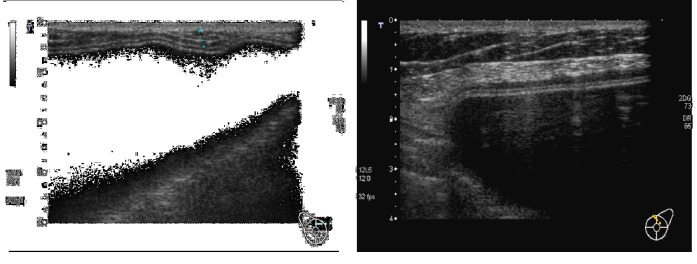
Biological membrane presenting as a hypoechoic periprosthetic layer at T0.

**Figure 2:**
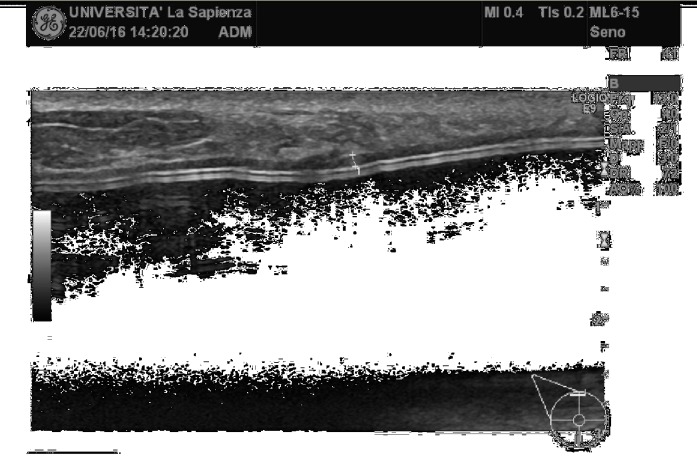
Biological membrane presenting as a hypoechoic periprosthetic layer at T0.

**Figure 3:**
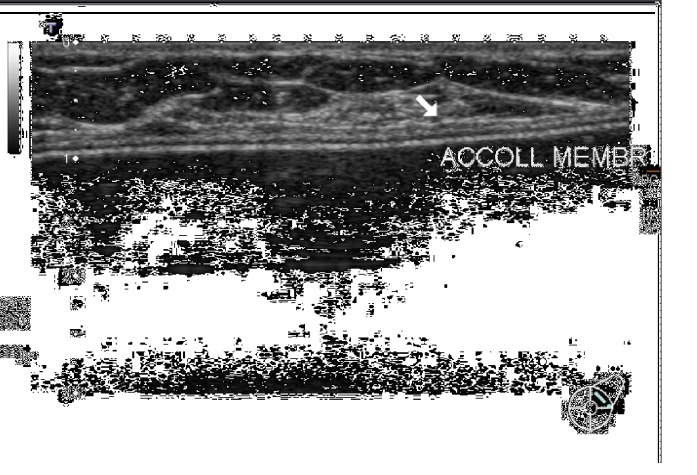
The biological membrane is partially visible at T2 because of its physiological reimbursement.

**Figure 4:**
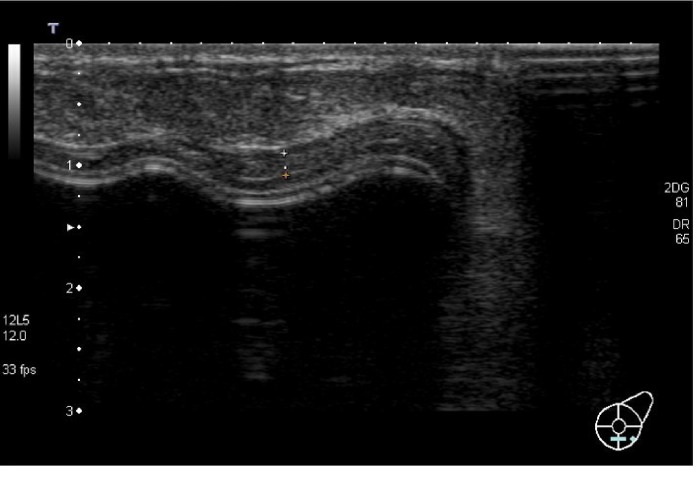
The biological membrane is still visible at T2 with a thickness of 2 mm, after radiation therapy.

Considering ADM visibility and thickness, the biological membrane was always visible at T0 as a hypoechoic periprosthetic layer measuring less than a millimeter in thickness (0.3-0.6 mm) (Figures 1-2); at T1, it was still partially visible in 10 patients, while at T2 and T3, it was not still identifiable (Figure 3). This is probably due to the high biocompatibility of the biological membrane, causing a mild fibroblastic reaction with focal tissue integration of the matrix and thus appearing less visible in course of follow-up. Only in one patient the biological membrane was more visible at T2 with a thickness of 2 mm; this woman was subjected to a recent radiation therapy on the breast implant (Figure 4).

In our series we did not observe cases of capsular contracture or rupture.

In most patients, breast prosthesis had regular morphologic aspect with lobulated margins.

In 18 patients, we observed that the biological membrane was well stretched along the convexity while relaxed at the medial and lateral side of the implant. In these points, the membrane partially folded and made some curves. This feature is well appreciable in the earlier evaluations probably for the presence of periprosthetic fluid, but over time, membrane folds can merge and stick together, thus appearing sometimes a focal hypoechoic lesion contiguous with the implant profile. The retrospective assessment of this finding allowed us to correctly interpret its nature and to distinguish this normal evolution of the matrix integration by other findings suspicious for disease recurrence.

During US evaluations, we paid attention to periprosthetic structures too, evaluating the presence of periprosthetic fluid, inhomogeneities of soft tissues, liponecrosis, and local complications.

**Figure 5:**
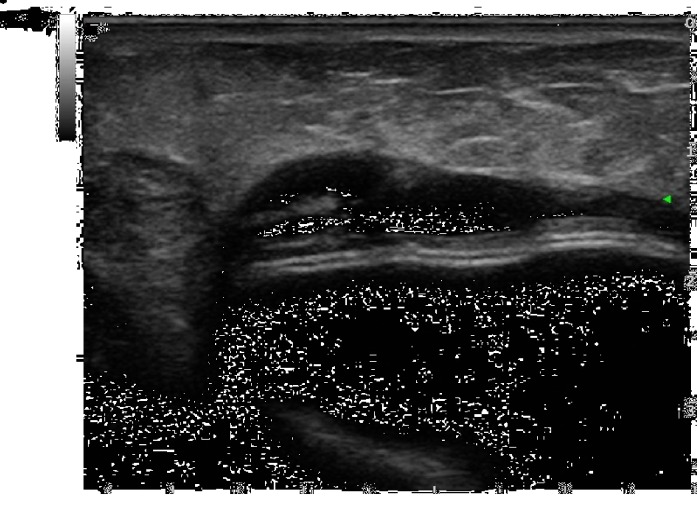
Marked periprosthetic fluid collection.

Very early postsurgery assessment, as it was proposed in our protocol, revealed in almost all patients the presence of periprosthetic fluid, sometimes a corpuscolated one. Fluid collections generally decreased over time and sometimes disappeared in the further assessments; only five patients still presented a thin and corpuscolated fluid layer at T3. Two patients had at T0 a marked periprosthetic fluid collection (40 cc and 30 cc, respectively), which decreased after a week of antibiotics to approximately 10 cc (Figure 5).

**Figure 6:**
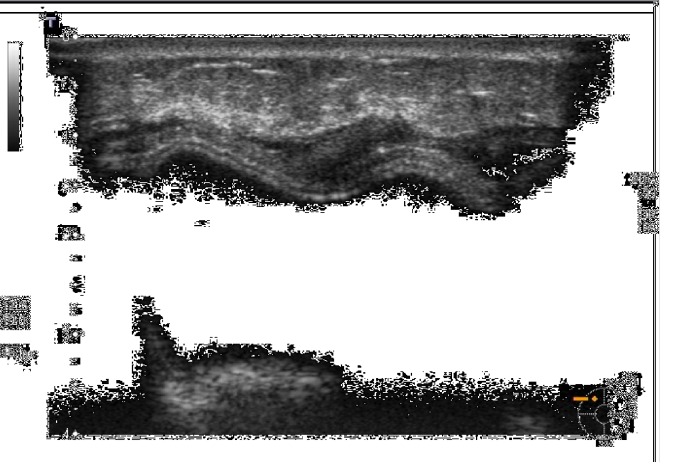
Inhomogeneity and thickening of the subcutaneous adipose tissue at T2, after radiation therapy.

In 20 patients, there was inhomogeneity and thickening of the subcutaneous adipose tissue in the early postsurgery evaluation. This finding was less significant in the successive US evaluations and finally disappeared. Just in one case, the same mentioned above, after radiation therapy, there was an important inhomogeneity of the breast soft tissue at T2 US examination (Figure 6).

**Figure 7:**
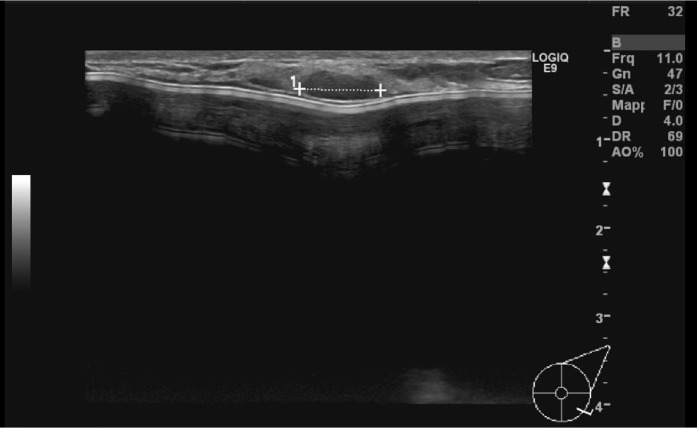
Hypoechoic nodule with well-defined margins in the inferior-external quadrant of the right breast that is a liponecrosis.

Liponecrosis was found in 10 patients in the US exams performed at T2 and T3, appearing as a hypoechoic nodule or mass with well-defined margins, ranging from 4 mm to 10 mm in size (Figure 7). Thanks to the strict follow-up, it was possible to observe and describe the natural history of this inflammatory process from the initial changes into fat tissue.

Another complication that we observed in immediate postoperative period was the occurrence of lymphocele, that is a collection of lymphatic fluid within the surgical site. Three patients had an axillary lymphocele in the US exams at T0 and T1.

**Figure 8:**
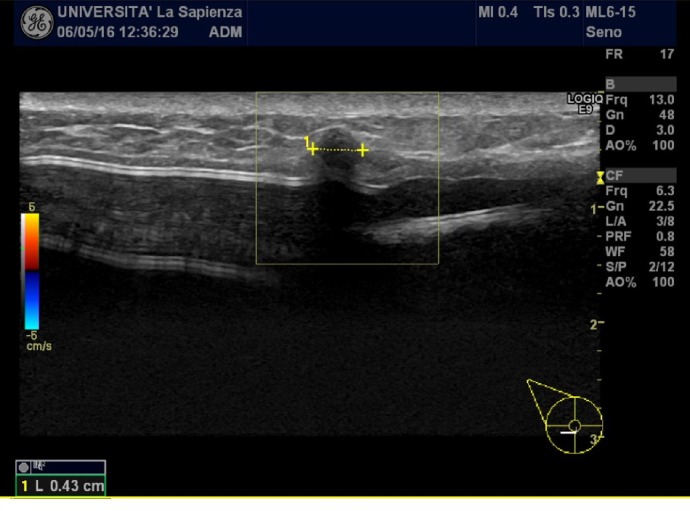
Hypoechoic nodule with irregular margins measuring 4 mm in the periareolar area on the right breast, suggestive for a suture granuloma.

At T0 US evaluation, we found a hypoechoic nodule with irregular margins measuring 4 mm in the periareolar area on the right breast, suggestive for a suture granuloma, in a patient who underwent bilateral mastectomy for ductal carcinoma in situ (Figure 8). At T1 US evaluation, it was still visible but decreased in size, measuring 2 mm.

A young woman with BRCA-1 mutation who underwent bilateral prophylactic mastectomy reported red, hot, and painful breast inflammation and burning sensation on inframammary folds one month after surgery. She underwent a breast MRI that revealed bilateral breast implant infection, treated with antibiotics and resolved in a month.

**Figure 9:**
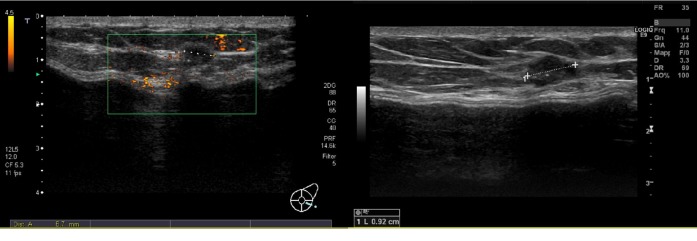
Hypoechoic nodule measuring 6 mm in the lower-internal quadrant, suggestive for a lump at T0 examination. At T1, this nodule occurred increasing in size measuring 9 mm, so a US-guided biopsy was performed.

**Figure 10:**
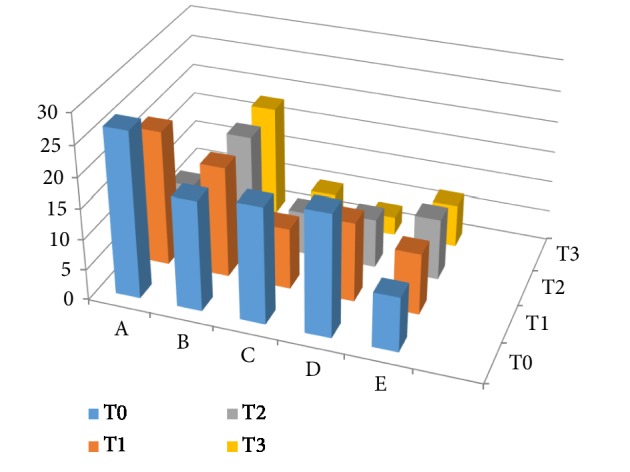
Graphic representation of the results of US evaluation over time, patients. A: membrane visibility; B: mediolateral membrane folds; C: periprosthetic fluid; D: inhomogeneity of soft tissues; and E: liponecrosis.

Finally, during a T0 US examination, we found a little hypoechoic nodule measuring 6 mm in the lower-internal quadrant suggestive for a lump, in a patient who underwent unilateral mastectomy and lymphadenectomy for invasive ductal carcinoma. At T1, this nodule appeared increasing in size measuring 9 mm, so a US-guided biopsy was performed; the histological report revealed that it was a residual disease (Figure 9) (Table 1 and Figure 10).

## 4. Discussion

Surgical options for breast reconstruction include alloplastic and autogenous reconstructions. In autologous approach, abdominal tissue is the gold-standard donor site. If the abdomen is not a suitable donor site, secondary donor sites such as the thigh or buttock are considered. Autologous tissues in breast reconstruction, however, involve the execution of generally more invasive interventions, a prolonged recovery (on average 5-7 days), and a longer postoperative rehabilitation [10].

Regarding alloplastic breast reconstructions techniques, ADM prosthesis was first described for use in breast surgery in 2001 by Dieterich et al. [11] and Salzberg [12].

Since then, this strategy gradually spread among reconstructive surgeons because of the better cosmesis in breast reconstruction, the reduction of late or irradiation-induced capsular contracture, and the improved aesthetic outcomes [13]. These advantages are particularly evident in prepectoral breast reconstruction in one-stage surgery technique. Among the “elderly” population, defined as those with a chronological age ≤ 65 years, breast cancer is largely represented. Although breast reconstruction in elderly patients is considered controversial, it is real part of the healing process, improving their quality of life; people are living longer and healthier and the survival rate for breast cancer is also improving. In these women, one-stage surgical option should be preferred because it is less invasive, allowing rapid recovery and prompt return to routinely activities. Among surgical techniques, the muscle-sparing Braxon wrap is the one that better satisfies these points [14]. Braxon non-cross-linked ADM allowed this new muscle-sparing technique that preserves the pectoralis major muscle. Maruccia et al. described that this surgical approach to Braxon ADM breast reconstruction involved skin- or nipple-sparing mastectomy that maintained a well-vascularized subcutaneous layer. The ADM edges were sutured together around the breast, placed into the subcutaneous breast pocket and then secured with apical, medial, and lateral absorbable stitches directly onto the pectoralis major muscle. One vacuum drain was inserted in the inframammary fold and removed between the seventh and tenth postoperative days, and the skin was closed in two layers. This surgical approach allowed reducing complications as fluid collection, promoting the early host integration of the matrix [15].

In last few years, ADM has been introduced into more than 60% alloplastic reconstructions in the United States, as reported by the American Society of Plastic Surgeons [16]. The increasingly use of ADM determines a consequent increasingly interest in describing the radiologic findings of ADM in postmastectomy reconstruction patients and poses a diagnostic challenge for Radiologists. Familiarity with the imaging features of ADM is essential for a correct diagnosis of normal matrix integration and of the possible complications, but to date, there is still a poor medical literature available on this topic [17].

Parvizi et al. first reported contrast-enhanced ultrasound evaluation (CEUS) to describe the vascularisation of ADM after implant-based breast reconstruction. They proved the “in vivo” evaluation of vascular ingrowth and tissue formation after breast reconstruction with ADM after follow-up of 1–18 months postoperatively in 16 patients [18].

In 2016, Seon Kim reported his experience of ultrasonography findings of AlloDerm in a patient who developed a palpable mass along the lower lateral profile of her reconstructed breast. The lesion did not show vascularity on color Doppler imaging. A left mediolateral oblique view (MLO) mammography and a simple chest radiography demonstrated a band-like lesion at the lower aspect of the reconstructed left breast. Nonenhanced computed tomography (CT) of the chest demonstrated an oval-shaped lesion with soft tissue density along the superficial aspect of the implant. After discussion with the plastic surgeon, this location and configuration was identified as consistent with the AlloDerm® sling used in reconstruction surgery (BI-RADS 2) [19].

In 2009, Buck et al. reported the case of a patient with a new palpable mass in her breast after mastectomy. After surgical excision, it was confirmed to be a foreign body giant cell infiltration, secondary to the ADM used in reconstruction [20].

All these experiences show how enhanced characterization of benign finding may help in distinguishing them from tumor recurrence or other foreign body.

As recently proved by Onesti et al. performing the biopsy of the periprosthetic tissue 12 months after surgery, histological examination showed the almost complete integration of ADM with the patient tissue. In the biopsy specimen, the matrix was crowded with lymphocyte, histiocytes, and vascular vessels containing erythrocytes. The immunohistochemical analysis conducted on the biopsy confirmed the presence of active proliferation within the matrix (Ki-67 positive cells), myofibroblast invasion (*α*-smooth muscle actin positive cells), and neovascular ingrowth (CD31 positive cells) [21].

These specific findings were confirmed in imaging by US examinations conducted in our study.

Despite some limits such as the small sample size and the short-term follow-up during only 12 months, in our experience the US evaluation and clinical follow-up allowed observing the natural postoperative changes in ADM prosthesis and becoming familiar with normal findings. This aspect is necessary in order to distinguish benign findings from malignant ones.

## 5. Conclusion

In conclusion, ADM has been shown as a safe option for women candidates for mastectomy. If Radiologists make experience in US examination during postsurgery follow-up, US technique can be a valid tool for the evaluation and the identification of both physiological and pathological findings, such as local surgery complications and recurrent disease.

## Figures and Tables

**Table 1:** Results of the study, patients.

	T0	T1	T2	T3

*Parameters*				
Visibility of the membrane	27	22	8	1
Mediolateral membrane folds	18	18	18	18
Periprosthetic fluid	19	10	7	5
Inhomogeneity of soft tissues	20	13	8	3
Liponecrosis	9	10	10	7
*Complications*				
Seroma	3	3	-	-
Suture granuloma	1	1	-	-
Nipple introflexion	-	-	1	-
Prosthetic infection	1	-	-	-
Residual disease	-	1	-	-

## Data Availability

The authors confirm that all data underlying the findings are fully available without restriction.
